# Associations between the METS-IR index and cognitive function in community-dwelling Chinese middle-aged and older adult individuals: a cross-sectional study

**DOI:** 10.3389/fpubh.2025.1607228

**Published:** 2025-07-29

**Authors:** Nian Jiang, Chenlu Ma, Zhenning Feng, Yongjun Tang, Xiaolong Chen, Yingxu He, Weiyi Pang

**Affiliations:** ^1^Guangxi Key Laboratory of Environmental Exposomics and Entire Lifecycle Heath, Guilin Medical University, Guilin, China; ^2^School of Public Health, Guilin Medical University, Guilin, China; ^3^School of Humanities and Management, Guilin Medical University, Guilin, China

**Keywords:** METS-IR, cognitive impairment, middle-aged and older adult, insulin resistance, CHARLS

## Abstract

**Objective:**

The relationship between insulin resistance and cognitive function has long been a subject of interest, but the association between the metabolic syndrome-insulin resistance (METS-IR) index and cognitive impairment remains unclear.

**Methods:**

We utilized data from the 2015 China Health and Retirement Longitudinal Study (CHARLS) national survey, which, after screening, included a final sample of 12,307 participants. Cognitive function was assessed through face–to–face interviews via the MMSE scale. Multivariate logistic regression was used to evaluate the correlation between the METS-IR index and cognitive impairment. Using regression analysis results from fully adjusted models, we subsequently explored the nonlinear relationship between the METS-IR index and cognitive impairment via smooth curve fitting with constrained cubic splines and sought potential inflection points. Additionally, we executed a battery of sensitivity and subgroup analyses to validate the robustness of our findings.

**Results:**

The study included 12,307 participants, of whom 49.02% were aged 45–60 years and 52.89% were female. The results revealed that for each unit increase in the METS-IR index, the risk of cognitive impairment increased by 1.4% (*OR* = 1.014, *95% CI*: 1.004–1.023; *p* < 0.01). When the METS-IR index was used as a categorical variable, compared with Q1, the odds of cognitive impairment increased by 17.1, 38.7, and 49.5% for each unit increase in the METS-IR index in the Q2, Q3, and Q4 groups, respectively. In addition, a nonlinear pattern was found in the analysis, and the endpoint of the METS-IR index was determined to be 38.1. On the left side of the endpoint, a one-unit increase in the METS-IR index was associated with a 3.1% increase in the risk of cognitive impairment. On the right side of the endpoint, the risk of cognitive impairment increased by 1.0% for each unit increase in the METS-IR index (all *p* < 0.05).

**Conclusion:**

This study highlighted the significant association between high METS-IR and the risk of cognitive impairment in Chinese middle-aged and older adult individuals. In addition, there was a specific nonlinear relationship between the METS-IR index and cognitive impairment (the inflection point was 38.1). Lowering the METS-IR index below 38.1 through lifestyle changes and diet control can significantly reduce the risk of cognitive impairment and may decrease the incidence of dementia.

## 1 Introduction

The worldwide phenomenon of population aging presents numerous challenges, particularly with respect to the growing concern over cognitive decline (1–3). Cognitive impairment typically signifies a reduction in an individual’s functional capacity and is considered a transitional phase between normal cognitive health and dementia (4). According to the latest World Alzheimer’s Disease Report, the number of people living with dementia globally is expected to double from 55 million in 2019 to 139 million by 2050. In China, the incidence and mortality rates of Alzheimer’s disease are increasing rapidly, making it the fifth leading cause of death for both urban and rural residents (5). Currently, there is a lack of treatments that can effectively slow the progression of dementia, which further highlights the importance of early recognition of cognitive impairment. Prompt interventions, such as cognitive rehabilitation therapy, dietary modification, and moderate-intensity exercise, can substantially reduce the risk of dementia (6).

Metabolic syndrome poses a significant risk for cardiovascular disease, affecting nearly one-third of adults in China and often cooccurring with obesity and type 2 diabetes (7). Insulin resistance, characterized by the reduced effectiveness of insulin in target organs, is regarded as the central mechanism underlying metabolic syndrome. Recent research has identified infrared heat not only as an early indicator of type 2 diabetes but also as a novel risk factor for cognitive decline, impacting both diabetic and nondiabetic patients (8). The existing evidence indicates that insulin not only crosses the blood–brain barrier via relevant receptors to play a key role in regulating behavior and metabolic processes but also influences memory formation by modulating the *β*-amyloid burden and synaptic plasticity (9). There are various methods for clinically assessing insulin resistance (IR), with the insulin clamp and glucose tolerance tests widely recognized as frequently used standards for measuring peripheral insulin sensitivity. Nevertheless, its application in routine clinical practice is limited because of its complex procedures and invasive nature (10). Therefore, there is an urgent need for an efficient and convenient diagnostic method to assess IR levels in patients.

Research has demonstrated that the METS-IR index, which integrates body mass index (BMI), triglyceride (TG), fasting plasma glucose (FPG), and high-density lipoprotein cholesterol (HDL-C) levels, offers a straightforward, reproducible, and reliable approach for assessing IR (11–13). Compared with traditional indicators such as fasting glucose and HOMA-IR, the METS-IR index has superior predictive performance because of its ability to capture multiple integrated clinical variables, including BMI, fasting blood glucose, and lipid profiles, simultaneously. This suggests that it may provide a more comprehensive assessment of IR. In patients with type 2 diabetes, METS-IR performs better than HOMA-IR does, and in the risk assessment of nonalcoholic fatty liver disease, METS-IR is superior to HOMA-IR in predicting incident cases (11, 14). Numerous epidemiological studies have consistently shown a strong association between METS-IR and various health conditions, such as cardiovascular disease, cancer, and other disorders (15–17). In a community-based population survey, the METS-IR index was used to identify patients with early mild cognitive impairment but only in patients without diabetes (18). However, the utility of the METS-IR as a proxy for IR in evaluating its link with cognitive decline remains limited, and no studies have explored its nonlinear relationship. Additionally, variations in study design, including differences in timing, METS-IR range, sex ratios, and adjustment factors, have led to an unclear understanding of the relationship between METS-IR and cognitive impairment in Chinese populations.

In this context, we hypothesized that the increase in the METS-IR index may be associated with cognitive impairment in middle-aged and older adult Chinese individuals. To verify this hypothesis, this study utilizes the China Health and Retirement Longitudinal Study (CHARLS) database—a nationwide, representative high-quality dataset on health status among middle-aged and older adult individuals. It focuses on relevant data from cognitive function surveys with the aim of exploring the relationship between the METS-IR index and cognitive impairment in this population.

## 2 Materials and methods

### 2.1 Study population

**Figure 1:**
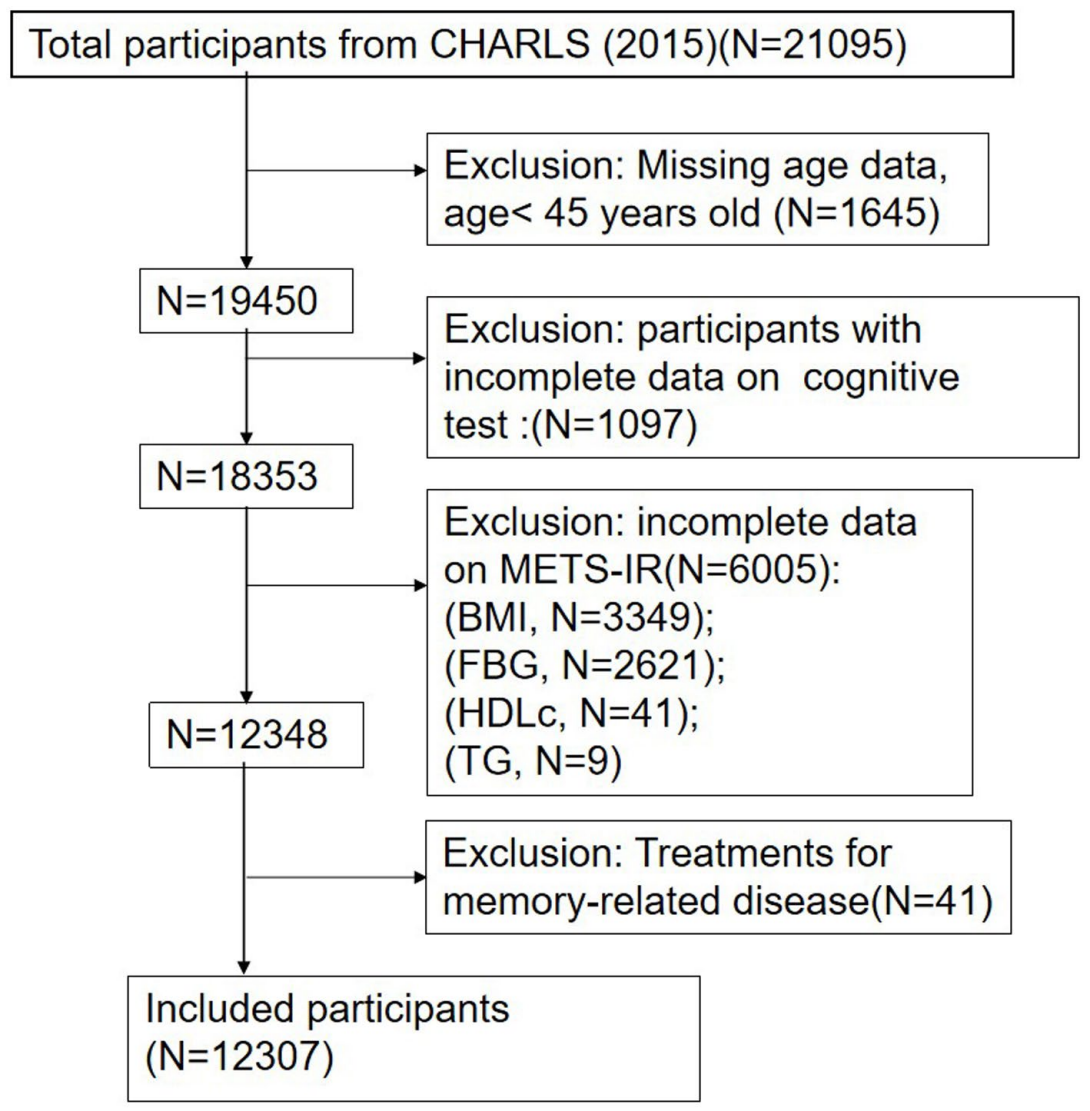
Participant selection process flowchart.

The cross-sectional data for this study were derived from CHARLS, a national longitudinal survey that employs a multistage probability proportional to the size sampling method. This method selected residents aged 45 years and older from 450 villages across 150 counties in 28 provinces of China for ongoing follow-up. The CHARLS comprehensively covers family, health, and various healthcare-related information, providing valuable insights into the health and well-being of China’s older adult population. The baseline survey commenced in 2011 with 17,708 participants, and follow-up surveys were conducted biennially, resulting in five waves of available data (2011, 2013, 2015, 2018, and 2020). Blood samples were collected during the 2011 and 2015 surveys. This study utilized data from the third wave of the 2015 survey, with a total sample size of 21,095 participants. During the data preprocessing phase, 1,645 participants younger than 45 years old and 1,097 individuals with incomplete cognitive data were excluded. Further screening excluded 6,005 participants lacking relevant indicators of the METS-IR index and 41 participants who had taken psychotropic medications related to memory. Ultimately, a total of 12,307 participants were included in the analysis. The selection process for participants is detailed in Figure 1. All participants provided informed consent prior to their involvement, and the study received ethical approval from the Peking University Biomedical Ethics Review Board (IRB 00001052-11015).

### 2.2 Assessment of cognitive function

In the CHARLS, cognitive function was assessed via the Mini-Mental State Examination (MMSE) questionnaire, a well-established tool that evaluates cognitive performance through tests of episodic memory and mental integrity. Episodic memory was assessed via two methods: immediate recall and delayed recall. For the immediate word recall test, participants were asked to memorize a list of 10 words and recall as many as possible. After a 5-min interval, they were then given a delayed word recall test, where they were asked to repeat the same words. Each correctly recalled word was scored, with a total possible score of 20 for overall situational memory.

The assessment of mental integrity included tests of temporal orientation, numerical ability, and figure-drawing skills. For temporal orientation, participants were asked to provide the survey date (including month, day, and year), day of the week, and season. The digit span test required participants to serially subtract 7 from 100 five times. Additionally, participants were asked to copy an image of two overlapping pentagrams. Like in the episodic memory test, each correctly answered question or successfully copied image earned one point, with a total score for mental integrity ranging from 0 to 11. The scores for episodic memory and mental integrity were combined to form an overall cognitive score, which ranged from 0 to 31. Previous research has confirmed that the use of the MMSE scale to assess overall cognitive function in middle-aged and older adult Chinese individuals has satisfactory validity and reliability (19). Cognitive impairment is typically defined as an overall cognitive score less than 11 (20, 21).

### 2.3 Definition of METS-IR

In this nationwide survey, blood samples from participants were collected by the Chinese Center for Disease Control and Prevention. All personnel involved in sample collection underwent rigorous training, passed examinations, and met strict qualification standards. The collected blood samples were transported to a local laboratory and stored at 4°C. Venous blood was separated into plasma and red–brown layers, with each component stored in separate 0.5 mL cryotubes. The cryotubes were immediately refrigerated at −20°C and shipped to the Chinese Center for Disease Control and Prevention headquarters in Beijing within 2 weeks. Upon arrival, they were placed in deep freezers and maintained at −80°C until analysis at the CMU laboratory. Notably, in the third wave of the CHARLS, the target sample for blood samples was a total of 21,100 interviewed individuals. In practice, blood samples were collected from 13,420 participants, resulting in a response rate of 64%. Data on FPG, TG, BMI, and HDLc were collected to calculate the METS-IR index as follows: METS-IR = (ln [(2 × FPG) + TG] × BMI)/(ln [HDLc]) (16). The participants were divided into four groups according to the quartile of the METS-IR index (Q1, Q2, Q3, and Q4).

### 2.4 Covariates

In accordance with previous studies (6, 17), demographics and health-related factors were considered common variables in the analysis of this study. The demographic variables included gender (male and female), age (45–60 years, 60 years and older), marital status (married and cohabiting, others including divorced, widowed, unmarried and separated), education (illiterate, primary school, secondary and above) and place of residence (urban, rural). The health-related variables included smoking status, alcohol consumption, body mass index, hypertension, diabetes, depression, etc. Smoking status was classified as never smoked, current smoker, or former smoker. Drinking conditions are defined as drinking at least once a month and are classified as never drinking alcohol, drinking less than once a month, and drinking more than once a month. BMI was calculated as weight (in kilograms) divided by the square of height (in meters), with units of kg/m^2^. BMI falls into three categories: less than or equal to 18.5, 18.5–24, and more than or equal to 24. Hypertension was defined as blood pressure above 140/90 mm Hg (based on the average of three measurements), self-reported hypertension or the use of antihypertensive medication by participants. Diabetes was defined as a fasting blood glucose greater than or equal to 7.0 mmol/L or self-reported diabetes, with the use of antidiabetic medications. The CHARLS assesses participants’ depression symptoms via the 10-item Center for Epidemiologic Studies Depression Scale (CESD-10), which has proven to be an effective, reliable, and practical tool for assessing mental health (22). In accordance with previous studies, participants who scored 10 or more on this scale were defined as having depression (23).

### 2.5 Statistical analysis

Continuous variables are shown as the means ± standard deviations (SDs), whereas categorical variables are presented as counts (percentages). Differences across the various quartile groups of METS-IR were evaluated via the chi-square test or the Kruskal–Wallis H test. The participants were categorized on the basis of METS-IR quartile, and the baseline characteristics of each group of individuals were compared.

First, a multivariate logistic regression model was used to assess the correlation between METS-IR and cognitive impairment. Three models were developed: Model I was not adjusted; Model II was adjusted by sex and age; and Model III was fully adjusted to include factors such as sex, age, marital status, education, location, smoking status, drinking status, BMI, hypertension, diabetes, and depression.

Second, the potential nonlinear relationship between METS-IR and cognitive impairment was investigated via a constrained cubic spline model based on the Model III adjustment variable. Likelihood ratio tests were also used to determine threshold effects between the METS-IR index and cognitive impairment and to identify potential inflection points in the case of nonlinear associations. Moreover, saturation threshold effect analysis was employed to identify potential inflection points.

Subsequently, subgroup analysis and interaction analysis were performed according to sex, age, marital status, education level, smoking status, alcohol consumption, BMI, and depression status to investigate whether these factors affect the relationship between METS-IR and the risk of cognitive impairment.

Furthermore, previous studies have shown a significant association between hypertension, diabetes, overweight and obesity, and cognitive impairment (24–26). Several sensitivity analyses were conducted to verify the robustness of these results. First, participants with no hypertension were analysed. Second, the association between METS-IR and cognitive impairment was explored in participants without diabetes. Finally, participants with a body mass index above 24 were excluded from the sensitivity analyses.

We used R software (version 4.3.3) and SPSS (version 26.0) for all the statistical analyses, and *p* < 0.05 (two-tailed) was considered statistically significant.

## 3 Results

### 3.1 Baseline characteristics of participants based on MetS-IR quartiles

This study included a total of 12,307 participants, 52.89% of whom were female and 47.11% of whom were male. Participants aged 45–60 years accounted for 49.02%, whereas those aged 60 years and above accounted for 50.98%. The participants were divided into four groups based on the METS-IR quartile: Q1 (< 30.41 mg/dL), Q2 (30.41–35.97 mg/dL), Q3 (35.97–41.12 mg/dL), and Q4 (> 41.12 mg/dL). The means ± standard deviations of each quartile were 27.22 ± 2.34, 32.84 ± 1.36, 37.64 ± 1.50, and 46.06 ± 6.28, respectively. In the highest quartile (Q4), there was a greater proportion of female participants with hypertension, diabetes, overweight, and obesity than in the other groups. In contrast, the proportions of men in this group of smokers and alcohol consumers were lower. Additionally, participants with higher METS-IR levels tended to have elevated BMIs, TG levels, and TC levels. Furthermore, compared with those in the lowest quartile (Q1), participants in the other quartiles presented lower cognitive scores and tended to have a greater proportion of participants with cognitive impairment. Baseline characteristics are shown in Table 1.

**Table 1:** Baseline characteristics of the participants.

METS_IRquartile	Q1 (<31.41)	Q2 (31.41–35.97)	Q3 (35.97–41.12)	Q4 (>41.12)	*p* value
Participants (*n*)	3,081	3,074	3,078	3,074	
Age [years, mean (SD)]	62.86 ± 10.24	60.53 ± 9.66	59.99 ± 9.24	59.01 ± 9.12	<0.001***
Age-group (%)					<0.001***
45–60	1790 (58.10%)	1,488 (48.41%)	1,446 (46.98%)	1,308 (42.55%)	
> = 60	1,291 (41.90%)	1,586 (51.59%)	1,632 (53.02%)	1766 (57.45%)	
Gender (%)					<0.001***
Male	1,680 (54.53%)	1,425 (46.36%)	1,329 (43.18%)	1,363 (44.34%)	
Female	1,401 (45.47%)	1,649 (53.64%)	1749 (56.82%)	1711 (55.66%)	
Marital (%)					<0.001***
Married	2,585 (83.90%)	2,643 (85.98%)	2,681 (87.10%)	2,770 (90.11%)	
Other	496 (16.10%)	431 (14.02%)	397 (12.90%)	304 (9.89%)	
Education (%)					<0.001***
Illiterate	284 (9.22%)	228 (7.42%)	190 (6.17%)	177 (5.76%)	
Primary school	2,456 (79.71%)	2,496 (81.20%)	2,595 (84.31%)	2,608 (84.84%)	
Middle school and above	341 (11.07%)	350 (11.39%)	293 (9.52%)	289 (9.40%)	
Location (%)					<0.001***
City	541 (17.56%)	682 (22.19%)	905 (29.40%)	1,026 (33.38%)	
Village	2,540 (82.44%)	2,392 (77.81%)	2,173 (70.60%)	2048 (66.62%)	
Smoking status (%)					<0.001***
Never	1,537 (49.89%)	1842 (59.92%)	1968 (63.94%)	1921 (62.49%)	
Current	1,166 (37.84%)	882 (28.69%)	713 (23.16%)	666 (21.67%)	
Cessation	378 (12.27%)	350 (11.39%)	397 (12.90%)	487 (15.84%)	
Drinking status (%)					<0.001***
None of these	1838 (59.77%)	1951 (63.53%)	2054 (66.73%)	2,113 (68.85%)	
Drinking more than once a month	984 (32.00%)	830 (27.03%)	739 (24.01%)	705 (22.97%)	
Drinking less than once a month	253 (8.23%)	290 (9.44%)	285 (9.26%)	251 (8.18%)	
BMI group (%)					<0.001***
<18.5	684 (22.20%)	7 (0.23%)	0 (0.00%)	1 (0.03%)	
18.5–24	2,394 (77.70%)	2,512 (81.72%)	883 (28.69%)	84 (2.73%)	
≥24	3 (0.10%)	555 (18.05%)	2,195 (71.31%)	2,989 (97.24%)	
Hypertension (%)					<0.001***
No	2,382 (77.31%)	2,291 (74.53%)	2065 (67.09%)	1883 (61.26%)	
Yes	699 (22.69%)	783 (25.47%)	1,013 (32.91%)	1,191 (38.74%)	
Diabetes (%)					<0.001***
No	2,955 (95.91%)	2,873 (93.46%)	2,717 (88.27%)	2,313 (75.24%)	
Yes	126 (4.09%)	201 (6.54%)	361 (11.73%)	761 (24.76%)	
Depression (%)					<0.001***
No	1982 (64.33%)	2037 (66.27%)	2,115 (68.71%)	2,118 (68.90%)	
Yes	1,099 (35.67%)	1,037 (33.73%)	963 (31.29%)	956 (31.10%)	
Cognitive impairment (%)					<0.001***
No	1,436 (46.61%)	1,217 (39.59%)	1,053 (34.21%)	961 (31.26%)	
Yes	1,645 (53.39%)	1857 (60.41%)	2025 (65.79%)	2,113 (68.74%)	
Cognition score [mean (SD)]	10.71 ± 5.94	11.73 ± 5.65	12.53 ± 5.65	12.82 ± 5.51	<0.001***
SBP [mmHg, mean (SD)]	124.35 ± 20.31	126.21 ± 19.20	130.14 ± 19.32	132.89 ± 19.13	<0.001***
DBP [mmHg, mean (SD)]	72.25 ± 11.30	72.25 ± 11.30	76.82 ± 11.35	79.16 ± 11.59	<0.001***
BMI [kg/m^2^, mean (SD)]	19.80 ± 1.70	22.67 ± 1.46	24.94 ± 1.69	28.53 ± 4.05	<0.001***
FBG [mg/dL, mean (SD)]	93.09 ± 20.03	98.01 ± 25.06	104.26 ± 32.97	118.73 ± 49.34	<0.001***
TG [mg/dL, mean (SD)]	89.70 ± 38.81	117.18 ± 58.30	149.67 ± 77.85	216.91 ± 115.62	<0.001***
TC [mg/dL, mean (SD)]	180.61 ± 34.81	183.24 ± 35.93	185.22 ± 35.34	187.63 ± 38.91	<0.001***
LDLc [mg/dL, mean (SD)]	98.55 ± 28.50	104.79 ± 28.79	105.31 ± 28.00	100.72 ± 29.55	<0.001***
HDLc [mg/dL, mean (SD)]	60.35 ± 12.71	52.54 ± 9.28	48.56 ± 8.52	48.56 ± 8.52	<0.001***
METS-IR [mean (SD)]	27.22 ± 2.34	32.84 ± 1.36	37.64 ± 1.50	46.06 ± 6.28	<0.001***

SD, standard deviation; N, number; BMI, body mass index; SBP, systolic blood pressure; DBP, diastolic blood pressure; FPG, fasting plasma glucose; TG, triglyceride; TC, total cholesterol; LDLc, low-density lipoprotein cholesterol; HDLc, high-density lipoprotein cholesterol; METS-IR, index. *p < 0.05, **p < 0.01, ***p < 0.001.

### 3.2 Multiple regression analysis of the METS-IR index with cognitive impairment

The results of the multiple linear regression analysis of the correlation between the METS-IR index and cognitive impairment are shown in Table 2. METS-IR was significantly correlated with cognitive impairment, and subjects with a higher METS-IR index tended to have worse cognitive function. After full adjustment, the odds ratio suggests that for every unit increase in the METS-IR index, there is a 1.4% increase in the odds of cognitive impairment (*OR* = 1.014, *95% CI*: 1.004–1.023; *p* < 0.01). Furthermore, when the METS-IR index was used as a classification variable, a significant association was found between the quartile of the METS-IR index and cognitive impairment after sufficient adjustment of the variable. Compared with the first quarter, the risk of cognitive impairment was greater in the other quarters of participants (*OR* = 1.171, *95% CI*: 1.045–1.313, *p* < 0.01), (*OR* = 1.387, *95% CI*: 1.233–1.560, *p* < 0.001), and (*OR* = 1.495, *95% CI*: 1.321–1.692, *p* < 0.001), respectively. Furthermore, these values indicate that for each unit increase in METS-IR levels, the odds of cognitive impairment were increased by 17.1, 38.7, and 49.5% in the Q2, Q3, and Q4 groups, respectively, compared with those in the Q1 group.

**Table 2:** Association between the METS-IR index and cognitive impairment.

Exposure	*OR* (95%*CI*), *p*		
	Model I	Model II	Model III
METS-IR	1.034 (1.028–1.039) < 0.001***	1.028 (1.022–1.033) < 0.001***	1.014 (1.004–1.023) 0.005**
METS-IR quartile			
Q1	Reference	Reference	Reference
Q2	1.332 (1.204, 1.474) < 0.001***	1.243 (1.115,1.386) < 0.001***	1.171 (1.045, 1.313) 0.006**
Q3	1.679 (1.515, 1.861) < 0.001***	1.590 (1.424, 1.776) < 0.001***	1.387 (1.233, 1.560) < 0.001***
Q4	1.919 (1.730, 2.130) < 0.001***	1.715 (1.534, 1.919) < 0.001***	1.495 (1.321, 1.692) < 0.001***
*p* for trend	<0.001***	<0.001***	<0.001***

Model I: non adjusted. Model II: Adjusted for age and sex. Model III: Adjusted for age, sex, marital status, education, location, smoking status, drinking status, BMI, hypertension, diabetes status, and depression. OR, odds ratio; 95% CI, 95% confidence interval. *p < 0.05, **p < 0.01, ***p < 0.001.

### 3.3 Nonlinear relationship between the METS-IR index and the risk of cognitive impairment

**Figure 2:**
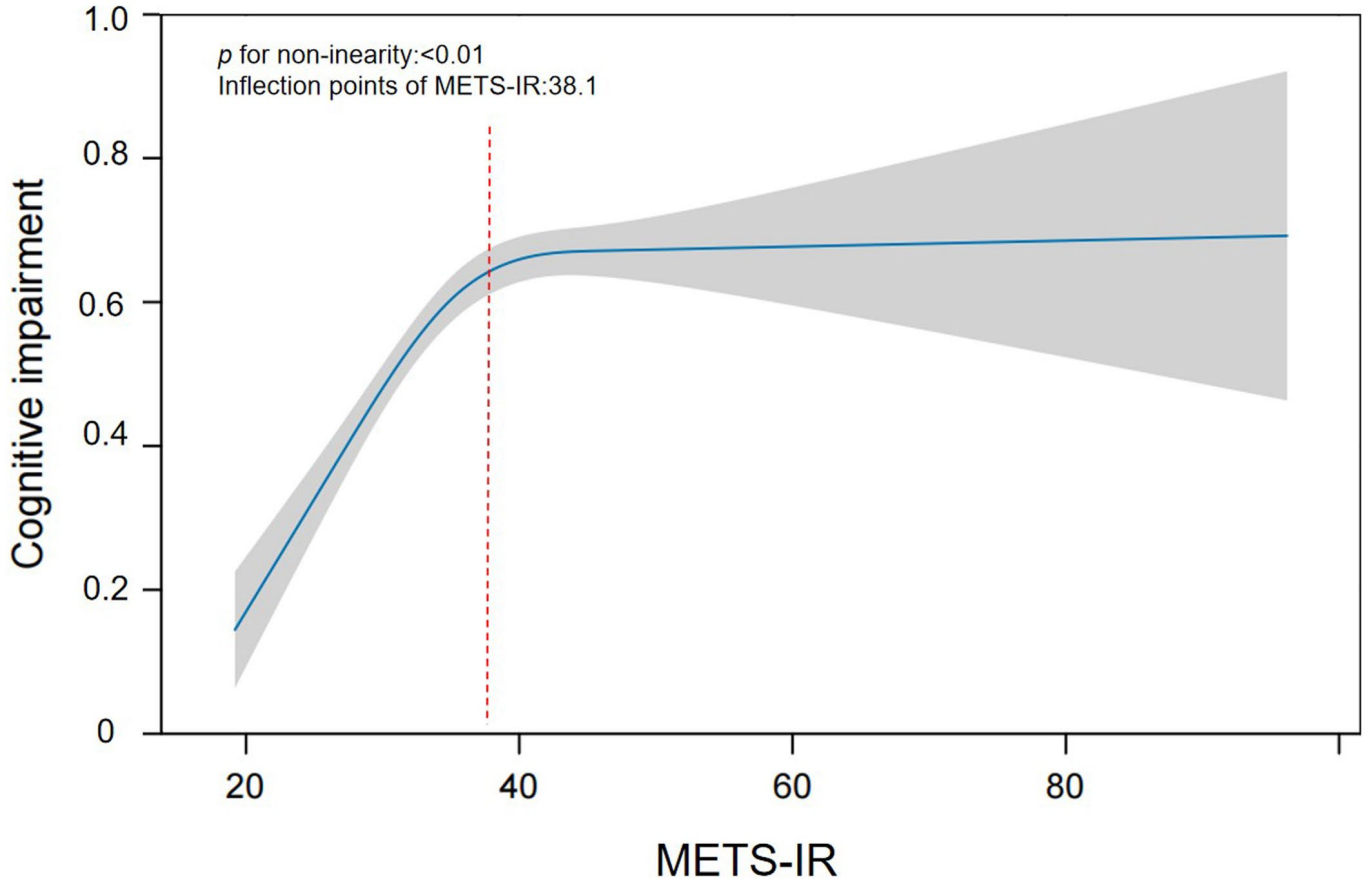
Smoothing curve fitting was used to assess the nonlinear relationship between the METS-IR index and cognitive impairment.

The nonlinear relationship between METS-IR and cognitive impairment risk is shown in Table 3. After fully adjusting for confounding factors, there was a special positive association between METS-IR and the risk of cognitive impairment. The inflection point is 38.1 according to saturation threshold effect analysis. Before the inflection point, (*OR* = 1.031, *95% CI*: 1.019–1.044, *p* < 0.001), and after the inflection point, (*OR* = 1.010, *95% CI*: 1.001–1.019, *p* < 0.05) are shown in Figure 2. To further validate the robustness of our findings, we included MMSE scores in our analysis. The results of the cognitive score were consistent with those of the cognitive impairment group, further demonstrating the reliability of the results (see Additional file 1).

**Table 3:** Threshold effect analysis of the METS-IR index on cognitive impairment based on Model III.

Outcome: cognitive impairment	*OR* (95%*CI*), *p*
Model I	
Fitting by the standard linear model	1.010 (1.003–1.017) 0.002**
Model II	
Inflection point	38.1
< Inflection point	1.031 (1.019–1.044) < 0.001***
> Inflection point	1.010 (1.001–1.019) 0.042*
Log likelihood ratio	<0.001***

OR, odds ratio; 95% CI, 95% confidence interval. *p < 0.05, **p < 0.01, ***p < 0.001.

### 3.4 Subgroup and sensitivity analysis

**Figure 3:**
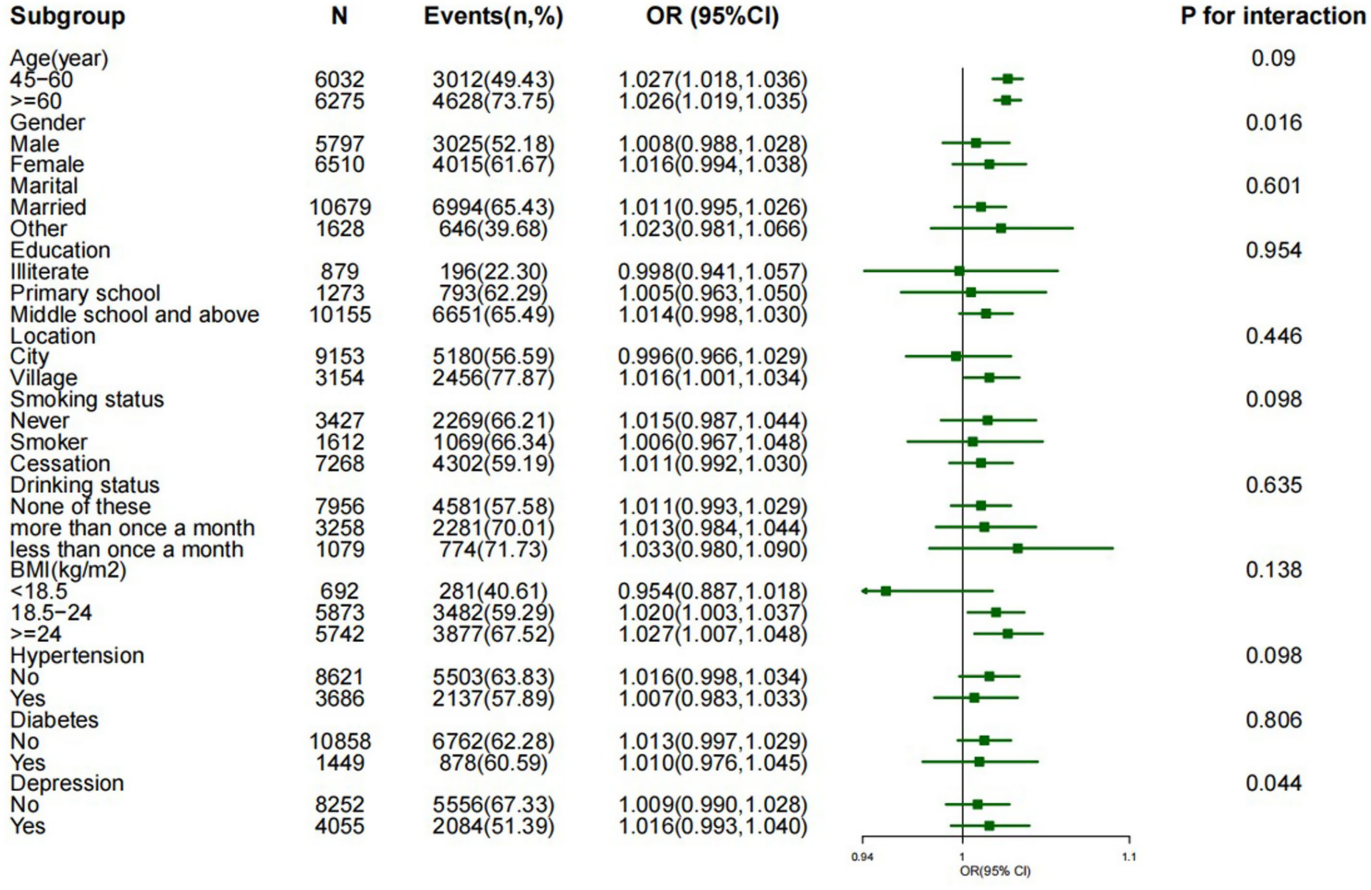
Effect size of the METS-IR index on cognitive impairment in the subgroup. Notes: adjusted for age, sex, marital status, education, location, smoking status, drinking status, BMI, hypertension, diabetes status, and depression.

The associations between METS-IR scores and cognitive impairment risk were not affected by age, marital status, education, smoking status, alcohol consumption, BMI, or depression, except for sex (*p* > 0.05 for interactions). In addition, female participants had a greater risk of cognitive impairment than male participants did (*p* = 0.016 for interaction), as shown in Figure 3. Sensitivity analyses were also conducted to ensure the reliability of the findings. We focused on specific groups and adjusted for various health factors to verify the strength and consistency of the relationship between the METS-IR score and cognitive impairment. First, the analysis was conducted in a nonhypertensive group of 8,621 participants, adjusting for age, sex, marital status, education, BMI, diabetes, depression, and other factors. The results still revealed a positive association between the METS-IR quartile and cognitive impairment (*OR* = 1.405, *95% CI*: 1.215–1.634; *p* < 0.001). Second, after excluding participants with diabetes, the results were similar after adjusting for confounding variables, and the association between the METS-IR quartile and cognitive impairment remained positive and significant (*OR* = 1.431, *95% CI*: 1.297–1.691, *p* < 0.001). Finally, the analysis was narrowed to participants with a BMI of less than 24 and was adjusted for all the previously mentioned factors. The results also revealed an association between the METS-IR quartile and cognitive impairment (*OR* = 1.342, *95% CI*: 1.106–1.904, *p* < 0.01) (see Additional file 2).

## 4 Discussion

This study was a cross-sectional study involving 12,307 participants to investigate the association between METS-IR and cognitive impairment in middle-aged and older Chinese individuals. The results revealed that elevated METS-IR was associated with a significantly increased risk of cognitive impairment. Furthermore, inflection points were determined by observing saturation threshold effects, and the relationship between METS-IR scores and cognitive impairment differed across tests. The findings remained uniform across all clusters and sensitivity examinations, particularly those focused on females and people aged 60 years and above.

As the world’s population ages, cognitive deficits intensify, making the related decline an acute public health issue. Symptoms of cognitive impairment usually occur several years before dementia begins, highlighting the importance of investigating the process and developing methods to prevent risk factors (27, 28). Previous studies have shown that genetic predisposition, cardiovascular disease, and other factors contribute to cognitive deterioration (29–31).

Insulin resistance is characterized by reduced sensitivity and responsiveness to insulin, which is at the core of various health problems, including diabetes, cardiovascular disease, and cognitive decline. Although the insulin clamp and fasting glucose have been considered the best standards for assessing IR, most studies tend to use alternative indicators for evaluation (32–34). A large cohort study revealed a significant association between the triglyceride glucose index, a simple measure of IR, and cognitive impairment among older adult individuals in China (35). Another study using HOMA-IR as a surrogate indicator for IR indicated that this index is associated with an increased risk of cognitive decline in obese adolescents and older individuals (36). In recent years, the METS-IR index has gained increasing interest as an important indicator for assessing IR due to its simplicity, reliability, and practicality compared with the HOMA-IR and insulin clamp techniques. Unfortunately, only one study has addressed this topic, which identified METS-IR as an effective predictor of mild cognitive impairment in nondiabetic individuals (18). Therefore, the current evidence regarding the relationship between METS-IR and cognitive impairment is quite limited, and further investigation is necessary to elucidate their association.

In this study, we revealed that there are sex differences in the risk of cognitive impairment, with further subgroup analysis revealing that women have a greater risk of cognitive impairment than men do. Research has indicated that women are more susceptible to cognitive impairment than men are, and the incidence of dementia is also higher among women across all age groups (2, 37). Research has suggested that estrogen is critical for memory function and that estrogen rehabilitation therapy can have a protective effect on neurons while improving cognitive performance (38). In this study, most female participants were in the perimenopausal or postmenopausal stage, which corresponds to a physiological phase characterized by a significant decline in estrogen levels within the body. This specific biological state can reasonably explain the more pronounced decline in cognitive ability observed in women than in men during our analysis. Therefore, the lack of protective effects of estrogen may play a dominant role in cognitive decline, while the relative importance of insulin resistance (IR) may be lower (2). However, the specific mechanisms by which estrogen affects cognitive function still require further investigation.

To further investigate the association between the METS-IR index and cognitive impairment in greater depth, we employed quartile classification of the METS-IR index prior to analysis. In Model III, after adjusting for potential confounding variables, we observed that an elevated METS-IR index was significantly associated with an increased risk of cognitive impairment compared with the lowest quartile (Q1) (*OR* = 1.495, 95% *CI*: 1.321–1.692, *p* < 0.001). Previous studies have identified blood pressure, diabetes, and obesity as risk factors for cognitive impairment (24–26). Therefore, we conducted a systematic sensitivity analysis by focusing on specific subgroups and adjusting for various health factors. The results confirmed a significant correlation between METS-IR and the risk of cognitive dysfunction. When we use smooth curve fitting to study the possible nonlinear relationship between two variables, we obtain interesting results. This study revealed a nonlinear relationship between METS-IR and cognitive impairment for the first time. The METS-IR inflection point was 38.1. When the METS-IR exceeded 38.1, each unit decrease in the METS-IR was associated with a 1.0% reduction in the risk of cognitive impairment. Conversely, when the METS-IR was less than 38.1, each unit decrease in the METS-IR corresponded to a 3.1% reduction in the risk of cognitive impairment. This suggests that as the METS-IR decreases, the risk of cognitive impairment gradually diminishes. The nonlinear relationship depicted in the curve-fitting plot aligns with these findings. For individuals with METS-IR values less than 38.1, the impact of other cognitive impairment risk factors is relatively diminished, thereby enhancing the effect of METS-IR. Upon examining the data, we observed that categorizing METS-IR, with the cut-off at Q4 (>38.1), places this threshold beyond the inflection point of the smoothed curve. When analysing the association between METS-IR and cognitive impairment risk, dividing the index into quartiles might diminish the statistical power and compromise result stability, primarily because very few participants present high METS-IR levels. Therefore, excessively high METS-IR values may weaken the direct impact on the increased risk of cognitive impairment when the model is constructed. This explains the observed nonlinear relationship between METS-IR and cognitive impairment risk. The discovery of this curve-like relationship has important clinical implications and can serve as a surrogate marker of IR to manage cognitive decline in middle-aged and older adult individuals. Reducing BMI, TG, and FPG levels through dietary modification, cognitive rehabilitation, and other comprehensive measures can significantly reduce the risk of cognitive impairment, particularly by maintaining the METS-IR below 38.1.

While the precise connection between cognitive decline and IR remains somewhat obscure, multiple elements account for these findings. Insulin passes through the blood–brain barrier via distinct receptors, affecting metabolic activity and behavior in the body (11, 39). IR often signifies a particular metabolic disorder characterized by elevated insulin levels, which may lead to neurodegenerative changes and persistent memory decline due to the long-term exposure of brain neurons to an environmental state of elevated insulin levels (40). Previous studies have suggested that peripheral insulin and brain insulin regulate glucose metabolism in the brain. Most individuals with IR/compensatory hyperinsulinemia exhibit metabolic abnormalities, which may decrease glucose metabolism in specific regions of the brain, subsequently affecting learning and memory by modulating synaptic plasticity, synapse density, synaptic transmission, and neurogenesis (41). In addition, impaired insulin signalling and neuroinflammation are both associated with cognitive function, and all of these pathological features are essential contributors to the decline in cognitive ability. Research has revealed elevated levels of inflammatory markers, particularly cytokines/chemokines involved in affected areas, in the brain tissue of patients with cognitive impairments (42). Animal experiments have revealed that in obese mouse models, the mechanism through which TNF-*α* is inhibited can effectively halt its interference in the insulin signalling process, thereby significantly improving insulin sensitivity and glucose homeostasis (43). In overweight and obese individuals, high levels of TG are more likely to cross the blood–brain barrier and may interfere with nerve transmission and lead to neurological dysfunction by inducing insulin receptor resistance (44). In the stratified analysis, the association between the METS-IR index and cognitive impairment was observed only in overweight and obese individuals, suggesting an association between traditional IR and obesity. Components of the METS-IR index, including FPG, TG, BMI, and HDLc, play a vital role in these mechanisms. Therefore, investigating these potential mechanisms simultaneously reveals a potential pathway between the METS-IR index and cognitive function.

With increasing age, the issue of cognitive decline has become increasingly prominent. Cognitive deficits, often seen as initial signs of dementia, are linked to significantly poor prognosis, impacting individual quality of life and imposing a burden on families and society (45). Therefore, early prevention of cognitive impairment is crucial and urgent. Our research revealed that the METS-IR index can be used as an alternative marker to predict cognitive impairment in middle-aged and older adult Chinese individuals and that there is a significant relationship between a high METS-IR index and cognitive impairment. Targeted interventions could help alleviate cognitive impairment, which may reduce the incidence of dementia. Additionally, investigating predictive risk is critical for early prevention to detect cognitive impairment.

This cross-sectional study is based on a representative sample from China and supports the hypothesis that there is a significant correlation between the METS-IR index and cognitive impairment. Unlike previous studies, our survey analysed the METS-IR index as a categorical and continuous variable related to cognitive impairment, reducing information loss and quantifying their relationship after adjusting for numerous confounding factors. In addition, the sensitivity study focused on participants without hypertension, with diabetes, or with a BMI less than 24 kg/m^2^. Further validation of the subgroup analysis confirmed that this relationship persisted in participants. In summary, we identified METS-IR as a risk factor for cognitive impairment and elucidated the relationship between the two, providing new perspectives for the prevention and management of cognitive impairment.

This study also has several limitations. First, given the cross-sectional nature of the study, the direct cause of the association between METS-IR levels and cognitive deficits is unclear. In addition, our analysis is based on existing databases, and there are minor data omissions in the calculation of the METS-IR index that may lead to selection bias in the results. Finally, as with all observational studies, despite adjustments for known potential confounding factors, residual confounding caused by unmeasured or uncontrolled confounding factors may still exist, so some unconsidered confounding variables may affect the results.

## 5 Conclusion

The study was conducted via the Chinese CHARLS database, which contains a nationally representative sample of Chinese middle-aged and older adult individuals. The study revealed a significant link between higher METS-IR levels and an increased risk of cognitive impairment. Notably, there is a nonlinear relationship between METS-IR and the risk of cognitive impairment. A further reduction in METS-IR was associated with a significantly reduced risk of cognitive impairment when the METS-IR value was less than 38.1. In addition, gender differences in the research findings were observed. This study provides additional insights into how cognitive function can facilitate clinical counselling and optimize prevention decisions. In the future, further prospective research is needed to gain a deeper understanding of the impact of insulin resistance on the risk of cognitive impairment and provide more effective guidance for the prevention and management of cognitive disorders.

## Data Availability

The datasets presented in this study can be found in online repositories. The names of the repository/repositories and accession number(s) can be found below: China Health and Retirement Longitudinal Study (CHARLS).
