# Supplementary material for: Associations between the METS-IR index and cognitive function in community-dwelling Chinese middle-aged and older adult individuals: a cross-sectional study
Source: Front Public Health. 2025 Jul 29;13:1607228. doi: 10.3389/fpubh.2025.1607228 (PMC12339461; doi:10.3389/fpubh.2025.1607228)

Additional file 1

**Table 1** Association between METS-IR index and cognitive scores

|  | *β*(95%CI), *p* |  |  |
| --- | --- | --- | --- |
|  | Model Ⅰ | Model Ⅱ | Model Ⅲ |
| METS-IR | 0.097(0.084-0.110)<0.001*** | 0.068（0.056-0.080）<0.001*** | 0.049（0.038-0.062）0.025* |
| METS-IR quartile |  |  |  |
| Q1 | Reference | Reference | Reference |
| Q2 | 1.025(0.741-1.310)<0.001*** | 0.669(0.407-0.931)<0.001*** | 0.474(0.226-0.721)<0.001*** |
| Q3 | 1.821(1.537-2.106)<0.001*** | 1.407(1.145-1.670)<0.001*** | 0.981(0.728-1.234)<0.001*** |
| Q4 | 2.116(1.831-2.400)<0.001*** | 1.464(1.120-1.728)<0.001*** | 1.046(0.784-1.308)<0.001*** |

Model Ⅰ: Non-adjusted

Model Ⅱ: Adjusted for age, gender

Model Ⅲ: Adjusted for age, gender, marital status, education, location, smoking, drinking, BMI, Hypertension, Diabetes, Depression.

*β*: Regression coefficients; 95%CI: 95% confidence interval

**p* < 0.05, ***p* < 0.01, ****p* < 0.001.

**Fig.1** The smoothing curve fitting was used to assess the nonlinear relationship between

METS-IR index and cognitive scores.


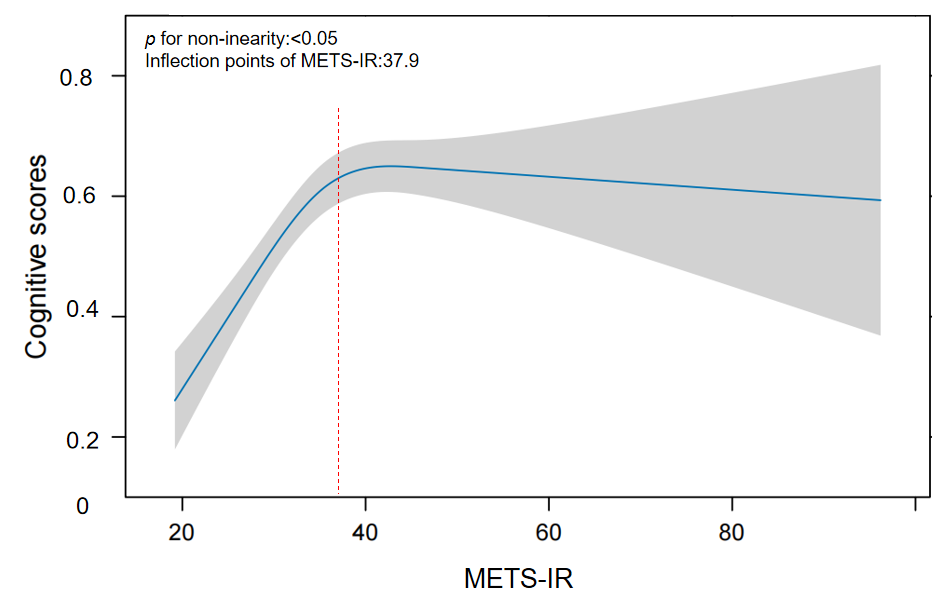

Supplement: Supplementary file 1 [file Supplementary_file_1.docx]
