# Supplementary material for: Associations between the METS-IR index and cognitive function in community-dwelling Chinese middle-aged and older adult individuals: a cross-sectional study
Source: Front Public Health. 2025 Jul 29;13:1607228. doi: 10.3389/fpubh.2025.1607228 (PMC12339461; doi:10.3389/fpubh.2025.1607228)
Supplement: Supplementary file 2 [file Supplementary_file_2.docx]

Additional file 2

**Table 1** Relationship between METS-IR and cognitive impairment in different sensitivity analyses

|  | OR (95%CI), *p* |  |  |
| --- | --- | --- | --- |
|  | Model Ⅰ | Model Ⅱ | Model Ⅲ |
| METS-IR | 1.008(1.004-1.019)0.012* | 1.014(1.003-1.025)0.013* | 1.026(1.013-1.040)<0.001*** |
| METS-IR quartile |  |  |  |
| Q1 | Reference | Reference | Reference |
| Q2 | 1.168（1.024-1.332）0.020* | 1.158(1.029-1.303)0.015* | 1.247(1.112-1.398)0.026* |
| Q3 | 1.429（1.240-1.638）<0.001*** | 1.366(1.208-1.545)<0.001*** | 1.360(1.155-1.603)<0.001*** |
| Q4 | 1.405（1.215-1.634）<0.001*** | 1.431(1.297-1.691)<0.001*** | 1.342(1.106-1.904)0.007** |

Model Ⅰ was a sensitivity analysis in participants without Hypertension (n=8621). Adjusted for age, gender, marital status, education, location, smoking, drinking, BMI, Diabetes, Depression.

Model Ⅱ was a sensitivity analysis in participants without Diabetes (n=10858). Adjusted for age, gender, marital status, education, location, smoking, drinking, BMI, Hypertension, Depression.

Model Ⅲ was a sensitivity analysis in participants with BMI<24 kg/m^2^ (n=6032). Adjusted for age, gender, marital status, education, location, smoking, drinking, Hypertension, Diabetes, Depression.

OR: Odds Ratio; 95%CI: 95% confidence interval.

**p* < 0.05, ***p* < 0.01, ****p* < 0.001.
